# Drought resistance of *Camellia oleifera* under drought stress: Changes in physiology and growth characteristics

**DOI:** 10.1371/journal.pone.0235795

**Published:** 2020-07-09

**Authors:** Xiaosan He, Linchu Xu, Chang Pan, Chun Gong, Yujuan Wang, Xinliang Liu, Yuanchun Yu

**Affiliations:** 1 Co-Innovation Center for Sustainable Forestry in Southern China, College of Biology and the Environment, Nanjing Forestry University, Nanjing, Jiangsu, China; 2 Jiangxi Academy of Forestry, Nanchang, Jiangxi, China; Hainan University, CHINA

## Abstract

To investigate the growth, physiological changes and mechanism of drought resistance of *Camellia oleifera* GWu-2 under drought stress conditions, changes in the main growth and physiological indices of GWu-2 under different water gradients were studied. Factor analysis was used to study the differences between indicators under different water gradients, and correlation analysis was implemented to analyze the relationship between different factors. We observed that the growth state, enzyme secretion, stomatal morphology and leaf osmotic adjustment substances were significantly affected by drought stress. In particular, increases in leaf abscisic acid (ABA), indole acetic acid (IAA) and methyl jasmonate (MeJA) contents under drought stress were negatively correlated with the stomatal opening degree, and the ratio of ZR/GA3 was significantly correlated with the growth and physiological indicators of GWu-2, indicating that different hormones respond differently to drought stress and have different functions in the growth regulation and drought resistance of GWu-2. We concluded that the drought resistance mechanism of GWu-2 was controlled by maintaining root growth to obtain the necessary water, increasing the contents of osmotic substances of leaves to maintain water holding capacity, reducing the transpiration of water by increasing leaf ABA, IAA and MeJA content to close stomata and reducing the damage caused by drought by increasing the activity of superoxide dismutase (SOD).

## Introduction

Drought stress is one of the most serious threats to plants in arid and semiarid regions [1]. Drought not only leads to declining average crop yield but also causes plant death in severe cases [2]. However, the responses vary from plant to plant under drought stress conditions, and many plants can grow well under such conditions, indicating that plants have strong drought resistance. Therefore, the study of physiological and ecological changes in plants under drought stress has become a prerequisite for drought-resistant crop cultivation.

Several scholars have conducted a large amount of research on the growth and physiological and biochemical characteristics of plants under drought stress as well as the drought resistance mechanism [3]. Numerous studies have shown that plants respond to drought threats by altering their metabolic rate, endogenous hormone content, carbon partitioning, and secretion of osmotic regulating substances [4,5], and plants respond differently to drought stress and thus exhibit different drought resistance [6–8]; thus, the mechanism of drought resistance changes between different plants [9].

In drought conditions, plants will exhibit a decreased growth index in terms of biomass, plant height, and root-crown ratio [10,11]. At the same time, certain changes will occur in the roots. The number of lateral roots will increase and become larger. This change in carbon partitioning of plants can ensure a relative reduction in above-ground biomass in the arid state, thereby reducing the water lost by transpiration, while the growth of lateral roots can increase the contact area between plants and soil, so that water in the soil can be better utilized [12].

As leaves are an important organ of photosynthesis, their external morphological characteristics can directly reflect the response of plants to drought stress, and these characteristics are closely related to the degree of water deficit [13,14]. Under drought stress, plant leaf stomata will close the first time to reduce water transpiration [15]. In addition, some adaptive changes will occur in the plant leaf structure, such as reduced leaf thickness and reduced leaf moisture content. These changes play important roles in enhancing water retention capacity, reducing transpiration, and improving photosynthesis [16].

Accumulating osmotic adjustment substances and improving antioxidant capacity are two important mechanisms for plants to survive under drought stress [6]. Under drought stress, plants will reduce their osmotic potential by accumulating osmotic adjustment substances such as Ca^2+^, valine, mannitol, betaine, soluble sugar, and soluble protein to regulate osmotic balance to prevent osmotic stress from causing damage [17,18]. In addition, drought stress signals can be transmitted to the entire plant through the synthesis and transmission of endogenous hormones and enzymes [3,19]. For example, plants can scavenge free radicals and protect their cells in arid environments by increasing the activity of enzymes such as superoxide dismutase (SOD), catalase (CAT), and ascorbate peroxidase (APX) [20,21]. At the same time, secretion of hormones such as abscisic acid (ABA), indole acetic acid (IAA), and zeatin nucleoside (ZR) will change accordingly. These are sensitive hormones in plants under drought stress and play important roles in regulating plant growth and development and enhancing drought tolerance [22].

Therefore, the drought resistance of plants is complex and involves several interacting properties, which are integrated in all aspects of plant growth and physiology. To understand the drought resistance mechanism and drought resistance of plants, it is necessary to conduct an experimental analysis of their physiological and growth elements.

*Camellia oleifera* is an important subtropical oil tree and an ecological tree in China. It is an economic tree species in the red soil hilly region of southern China. However, seasonal drought in the red soil region is severe, which has a great impact on the survival and growth of *Camellia oleifera* in this region, often restricting its production and causing economic losses. Gan Wu series *Camellia oleifera* is the main Camellia cultivar in Jiangxi Province. However, thus far, there have been few studies on the drought tolerance of Gan Wu series *Camellia oleifera*, which has limited its scientific planting and promotion. Consequently, GWu-2, a widely cultivated variety in the Gan Wu *Camellia oleifera* strain, was selected as the research object to study the drought tolerance and drought resistance mechanism.

The growth indicators are as follows: root length, number of lateral roots, root dry weight, above-ground dry weight, root-crown ratio, number of dead leaves, root-plant ratio, dry matter accumulation value, leaf thickness, upper and lower epidermis thickness, palisade tissue thickness, sponge tissue thickness, relative water content of leaves, stomatal length, stomatal width, and stomatal opening degree. Physiological indicators in leaves include soluble sugar (SS), soluble protein (SP), proline (PRO), superoxide dismutase (SOD), malondialdehyde (MDA), abscisic acid (ABA), indole acetic acid (IAA), zeatin nucleoside (ZR), Gibberellin (GA3), and methyl jasmonate (MeJA).

## Materials and methods

### Research area

The study area is located in the experimental site of Jiangxi Academy of Forestry, Nanchang City, Jiangxi Province, China, 12 km from the urban area, at 28°41′N, 115°48′E, 40 m above sea level, belonging to the mid-subtropical humid monsoon climate. The annual average temperature is 17.30°C, the monthly average temperature in July is 29.10°C, and the extremely high temperature is 40.60°C. The average annual rainfall is 1713.5 mm, and the annual average sunshine is 1778.6 h, greater than l0°C, and the accumulated temperature is 4480–4590°C.

### Materials

*Camellia oleifera* seedlings were 2-year-old cutting seedlings, and the cuttings were from the Germplasm Genebank of the Jiangxi Academy of Forestry. At the beginning of January 2016, 200 neat and disease-free Gwu2 *Camellia oleifera* seedlings were selected as test materials and planted in flower pots for normal cultivation in the open air for approximately 5 months. On the evening of July 7, 2016, all tested seedlings and the control group were water-drenched, and the water control test began on July 8. The diameter and height of the culture pot were 24.00 and 20.00 cm, respectively, and the potting soil was prepared by mixing red loam soil with chaff ash in a volume ratio of 5:1 (to improve the soil characteristics to be more conducive to the growth of *Camellia oleifera* seedlings). Each *Camellia oleifera* variety was treated 4 times, each treatment was repeated 3 times, and 12 *Camellia oleifera* seedlings were tested each time. At the same time, 4 pots of oil-free tea seedlings were set for each water gradient to determine the evaporation water consumption of the control groups.

### Moisture control

The preliminary experiment results of drought resistance of *Camellia oleifera* showed that when the soil water content was less than 20%, the leaves were slightly curled; when the soil water content was approximately 10%, the leaves appeared to be wilting, the growth of young leaves was affected, and some old leaves were shed; when the soil volume water content was less than 6%, the leaves of *Camellia oleifera* permanently wilted and fell off. Consequently, four moisture gradients were set, including a control group (with adequate water supply, W1), mild drought stress (W2), moderate drought stress (W3), and severe drought stress (W4).

On the evening of July 7, 2016, all experimental seedlings and the control group were permeated with water, and the water control test began on July 8. At approximately 8:00 am on July 10, 2016, the soil volumetric water content was measured with a soil hygrometer (a high-precision soil moisture measuring instrument, IMKO, Germany). If the soil volume water content was in the range of 20% to 36%, the current weight of the potted seedlings was recorded; if it was higher than 36%, the test was postponed until its water content was within the specified range; if it was lower than 20%, water was added as appropriate until the water content was within the specified range. The weight of the seedlings and their control group when the water content reached the prescribed water content was recorded as a basis for replenishing the water lost by transpiration every night from 20:00 to 22:00. The water contents of all the soil groups and the control group with sufficient water reached the set range on July 11.

After July 8, the drought stress group and the control group were no longer replenished with water. According to the water content measurement method and water control method of the normal water supply group, the water contents of the drought stress group and the corresponding control group were controlled according to mild, moderate and severe drought stress (Table 2). The soil moisture contents of each drought treatment group and its control group reached their specified soil moisture content ranges on July 17, July 19, and July 23, respectively. After reaching the experimental moisture setting, all groups were immediately sealed with plastic wrap to prevent evaporation of soil moisture. Water was added daily to keep the soil water contents of the experimental and control groups within the range set by the experiment. The measurement of various indicators officially started on July 26, and the test end time was September 26.

The soil properties of potted plants are shown in Table 1, and moisture control information is shown in Table 2.

**Table 1:** Physical properties and nutritional characteristics of experimental soil (g·kg^–1^).

Bulk density (g·cm^3^)	Organic matter (g·kg^–1^)	pH	Total nitrogen g·kg^–1^)	Total phosphorus (g·kg^–1^)	Total potassium (g·kg^–1^)	Available nitrogen (mg·kg^–1^)	Available phosphorus (mg·kg^–1^)	Available potassium (mg·kg^–1^)
1.42	39.20	3.95	1.19	0.57	17.30	121.00	28.40	193.00

**Table 2:** Soil water gradients of all treatments).

Moisture control	Field water holding capacity (%)	Soil mass water (%)	Soil volumetric water (%)	Average soil water (volume water content %)	Field water holding capacity (%)
W1 (adequate water supply)	39.00–73.00	14.00–26.00	20.00–36.00	25.26 ± 1.63	49.64 ± 3.20
W2 (mild drought stress)	23.00–37.00	8.00–13.00	12.00–18.00	14.35 ± 1.00	28.20 ± 1.96
W3 (moderate drought stress)	17.00–23.00	6.00–8.00	8.00–12.00	9.92 ± 0.40	19.49 ± 0.79
W4 (severe drought stress)	12.00–17.00	4.00–6.00	5.00–8.00	7.17 ± 0.20	14.09 ± 0.40

Average soil volume water content and field water holding capacity were obtained from several random samples in the corresponding process (n = 5).

### Methods

#### Determination of plant height and ground diameter

The plant height and ground diameter of all tested seedlings were measured at the beginning of the test (July 7, 2016) and at the end of the test (September 26, 2016). Plant height was measured with a steel tape measure, and ground diameter was measured with a Vernier caliper.

### Determination of biomass

Measurement of biomass was carried out at the beginning (July 7, 2016) and end of the test (September 26, 2016). At the beginning of the drought stress experiment, 4 to 5 seedlings with similar growth characteristics were selected to determine the dry weight of the whole plant. The roots, stems, and leaves of *Camellia oleifera* were separated at the end of the test, the total root length was measured with a tape measure to calculate the sum, and the total number of lateral roots was counted. Root fresh weight, root dry weight, leaf dry weight, and stem dry weight were determined by balance.

#### Determination of the number of dead leaves

At the beginning of the drought stress test, 3 *Camellia oleifera* seedlings of each treatment group (including the control group) were selected to determine the number of dead leaves. The number of dead leaves from each selected *Camellia oleifera* seedling that naturally fell from the beginning to the middle of the test was recorded.

Other indicators were calculated as follows:

Height-diameter ratio = plant height/ground diameter
Root-crown ratio = root dry weight/dry weight of above-ground part
Root-plant ratio = root dry weight/whole plant dry weight
Dry matter accumulation = biomass at the end of the test–initial biomass

#### Determination of structural characteristics of *Camellia oleifera* leaves

The leaf tissue was observed by electron microscopy. In the middle of the experiment, 3 pieces of mature leaves of *Camellia oleifera* shoots were collected, and the upper and lower parts of the leaves were sampled on the left and right sides. The area was approximately 2 mm × 0.5 mm. Leaf samples were immediately placed in a 2.5% glutaraldehyde solution with 0.1 mol∙L^–1^ phosphate buffer (pH = 7.2), transferred to cleaned medical vials, and allowed to sink by vacuum evacuation. These were stored in a refrigerator at 4°C until use.

Electron microscopy of the leaf was processed according to the method of Ren [23]. The samples were washed several times in phosphate buffer and then dehydrated with different concentrations of ethanol, then dehydrated twice with 100% ethanol, and then placed in isoamyl acetate twice for 15 min each. After the treatment was completed, samples were placed in a critical point dryer for drying and conductive treatment. Finally, samples were observed using a scanning electron microscope to obtain data on thickness of leaf, thickness of upper epidermis, thickness of lower epidermis, palisade tissue thickness, and sponge tissue thickness.

The length and width of the stomata were measured and calculated according to the scanning electron micrographs of the lower epidermis of the leaves.

Stomatalopeningdegree=Stomatalopeninglength×Stomatalopeningwidth×π/4

#### Determination of osmotic adjustment and antioxidant substances in *Camellia oleifera* leaves

In the middle of the trial (August 25, 2016), three samples were selected for each treatment. A total of 10–15 mature leaves were collected from the directions of the east, west, south, and north of the sample and mixed, and the mixed samples were then stored in an ultralow temperature refrigerator. The malondialdehyde (MDA), soluble sugar (SS), proline (PRO), soluble protein (SP) contents and superoxide dismutase (SOD) activity in *Camellia oleifera* leaves was measured by the thiobarbituric acid method, anthrone colorimetric method, ninhydrin colorimetry, Maslan blue colorimetry, and nitrogen blue tetrazolium (NBT) method, respectively. Each experiment was repeated 3 times, and the averages were taken as the result.

#### Determination of endogenous hormones in *Camellia oleifera* leaves

The contents of abscisic acid (ABA), indole acetic acid (IAA), zeatin nucleoside (ZR), gibberellin (GA3), and methyl jasmonate (MeJA) in leaves were determined by enzyme-linked immunosorbent assay (ELISA) according to Wu [24]. Each test sample of 0.5 g of oil tea was weighed, an appropriate amount of the sample extract was added, and then the sample was ground into a homogenate under an ice bath, then transferred to a 10-ml centrifuge tube and placed in a refrigerator at 4°C for 4 hours. After freezing, it was centrifuged for 15 minutes, and the supernatant was taken. An appropriate amount of the extract was added to the precipitate and then returned to the refrigerator at 4°C for 1 hour. After centrifugation, the supernatant was combined, and the volume was recorded. The supernatant was passed through a C-18 solid phase extraction column and then transferred to a 5-mL centrifuge tube and blown dry with nitrogen. Finally, the methanol in the extract was removed, and the sample was diluted to the appropriate volume.

#### Data processing

All experimental results were averaged after removing the outliers. Statistical analysis and analysis of variance were performed with SPSS 22. ANOVA was used to analyze the differences between indicators under different water gradients (using SPSS 22). The graph was created using OriginLab 2016. The table was created using Word 2016.

## Results

### Effect of drought stress on growth characteristics of *Camellia oleifera*

**Fig 1:**
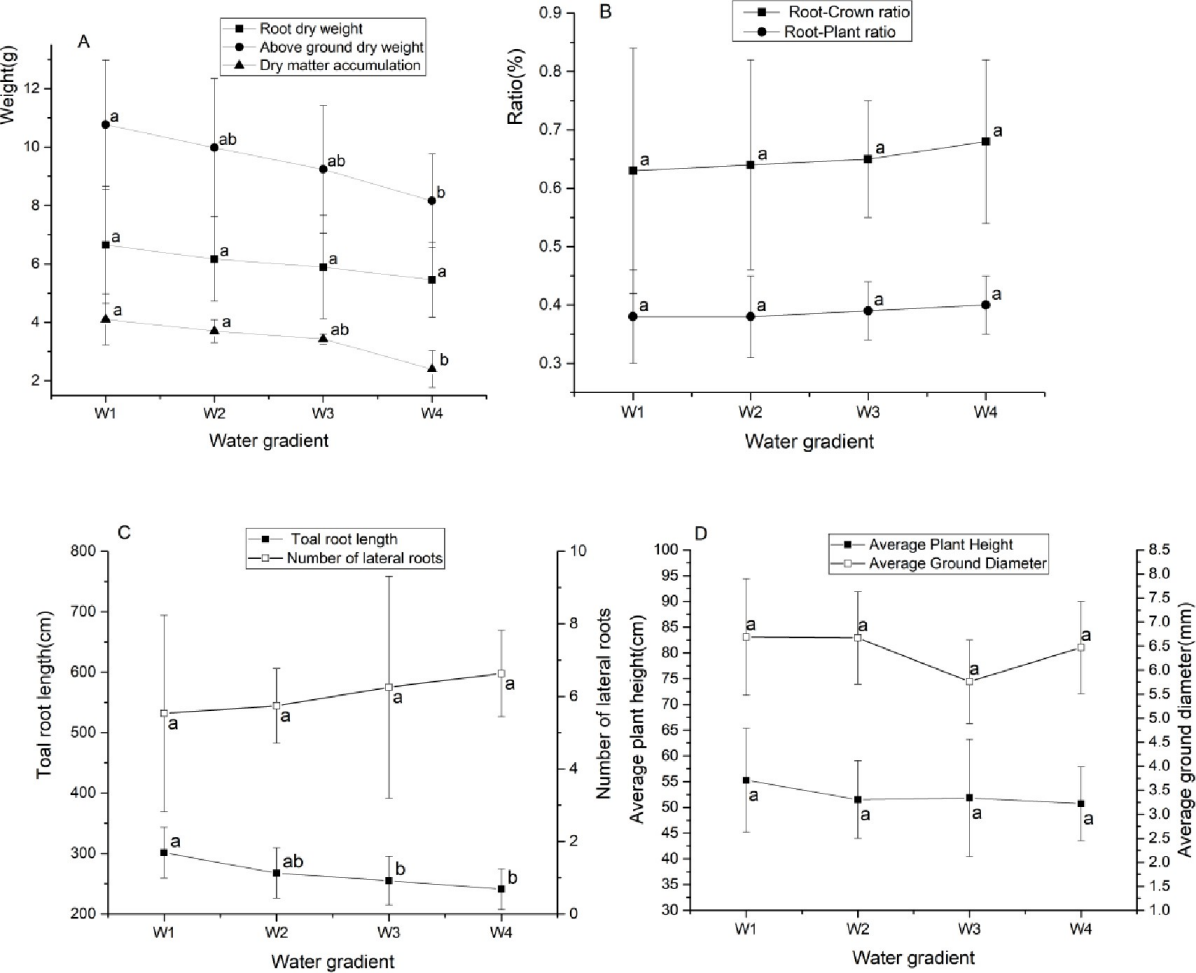
Growth of roots and biomass of *Camellia oleifera* under drought stress. W1: adequate water supply; W2: mild drought stress; W3: moderate drought stress; W4: severe drought stress. Each indicator value is the average value (Mean±SEs, n = 3). For the same type of observation indicators, different letters indicate statistically significant differences (p <0.05), same as below.

The total root length, above-ground dry weight and dry matter accumulation of GWu-2 were significantly decreased with the increased drought stress (Fig 1A and 1C; p<0.05). Compared with W1 treatment, the total root length decreased by 11.2%, 15.4%, and 20.0%, respectively. However, root dry weight and the number of lateral roots were not significantly affected by drought stress. Fig 1B and 1D show that the root-crown ratio, root-plant ratio, average plant height and average ground diameter were not significantly changed with the increased drought stress (p<0.05).

**Fig 2:**
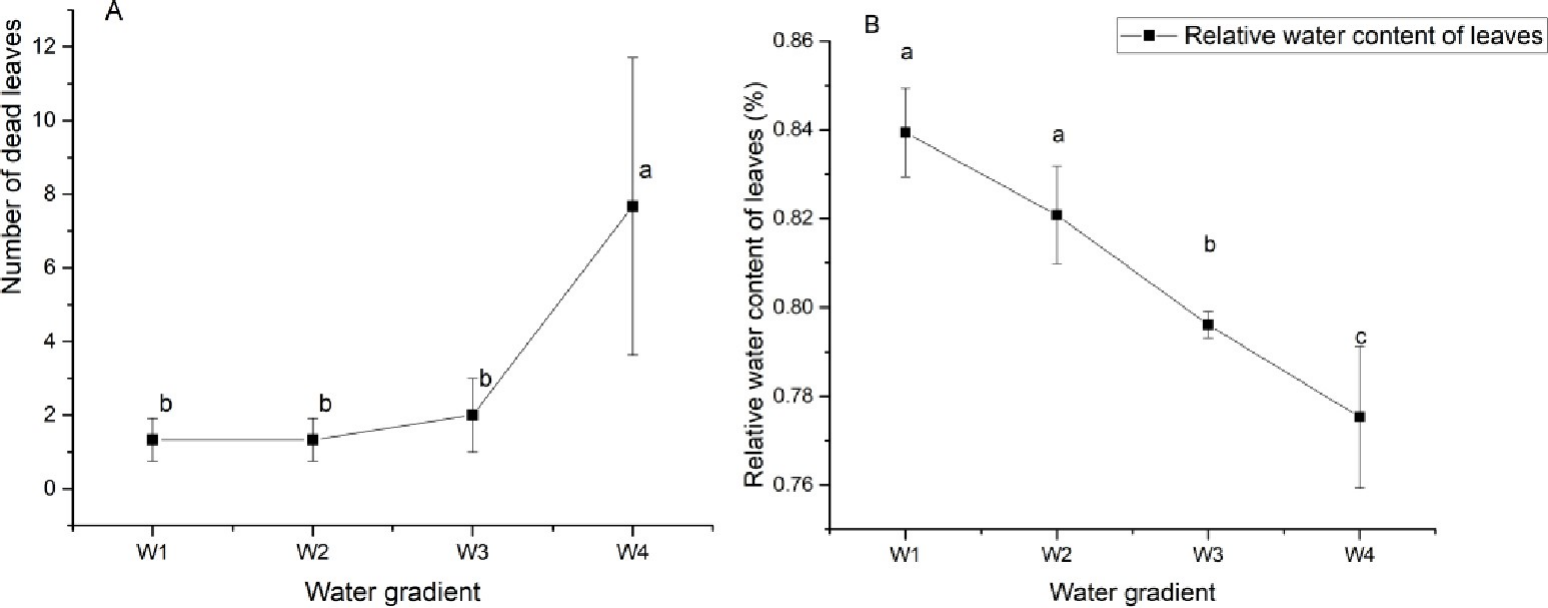
Effects of different degrees of drought stress on leaf characteristics of *Camellia oleifera*. W1: adequate water supply; W2: mild drought stress; W3: moderate drought stress; W4: severe drought stress. Each indicator value is an average value (Mean±SEs, n = 3).

Fig 2 illustrates that severe drought stress had a significant effect on the number of dead leaves and the leaf relative water content of GWu-2 (p<0.05). With the increased drought stress, compared with W1 treatment, the number of dead leaves increased 476.7%. Moreover, the relative water content of leaves decreased with increasing drought stress and decreased significantly under W3 and W4 treatments (p<0.05).

#### Correlation analysis between Camellia oleifera growth index and soil water content

Table 3 shows that the growth factors plant height, plant height growth, ground diameter growth, height-diameter ratio, total root length, root dry weight, above-ground dry weight, terminal biomass, and dry matter accumulation of GWu-2 had positive correlations with soil water content, and the numbers of lateral roots, root-crown ratio, root-to-plant ratio, and number of dead leaves were negatively correlated with soil water content. In addition, there were significant positive correlations between soil water content and plant height growth, height-diameter ratio, total root length, and root dry weight.

**Table 3:** Correlation analysis results of growth indices of *Camellia oleifera* and soil water contents under different soil water gradients.

Index	pH	GD	H-D ratio	PHG	NTR	NLR	RDW	ADW	R-C ratio	R-P ratio	Biomass	DMA	NDL
Water gradient	0.94	0.55	0.99**	0.99**	0.99**	–0.91	0.97*	0.94	–0.8	–0.81	0.95	0.85	–0.65

PH = plant height; GD = ground diameter; H-D ratio = height-diameter ratio; PHG = plant height growth; NTR = number of total roots; NLR = number of lateral roots; RDW = root dry weight; ADW = above-ground dry weight; R-C ratio = root-crown ratio; R-P ratio = root-plant ratio; DMA = dry matter accumulation; NDL = number of dead leaves.

*Correlation is significant at the 0.05 level (2-tailed).

**Correlation is significant at the 0.01 level(2-tailed).

#### Effects of drought stress on the stomatal characteristics of *Camellia oleifera* leaves

**Fig 3:**
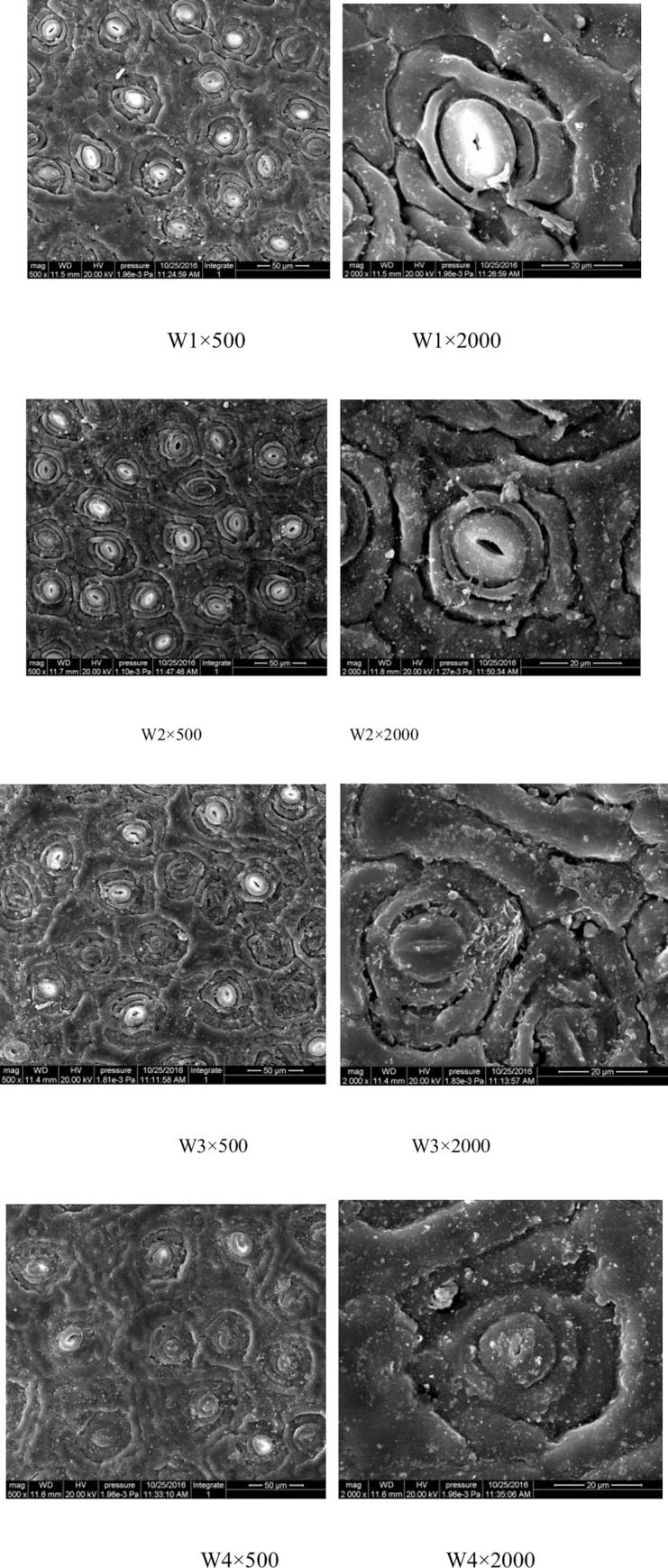
Electron microscope images of stomatal characteristics of *Camellia oleifera* leaves under different water gradients.

**Fig 4:**
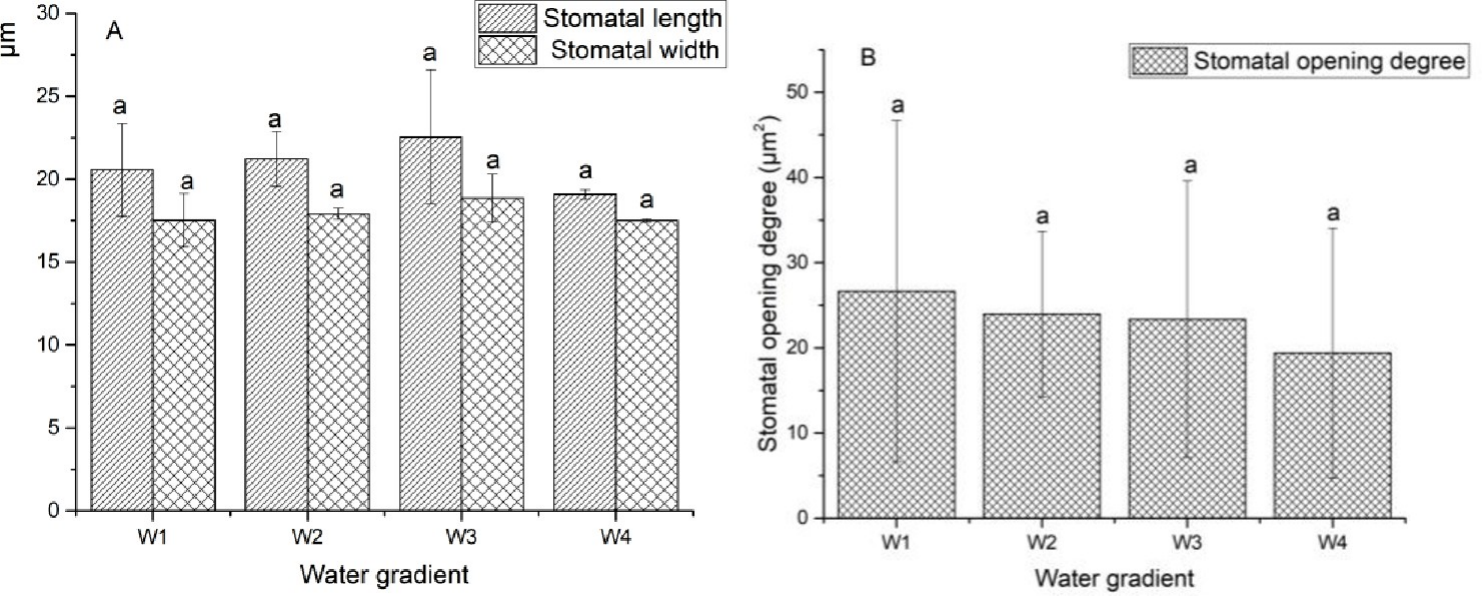
Trends of stomatal characteristic values of *Camellia oleifera* leaves under different water gradients. W1: adequate water supply; W2: mild drought stress; W3: moderate drought stress; W4: severe drought stress. Each indicator value is an average value (Mean±SEs, n = 3).

Fig 3 indicates that the stomatal opening degree gradually decreased as the degree of drought increased. Under severe stress, the stomata of GWu-2 were damaged and subsided, and the epidermal cells became dried. However, Fig 4 illustrates that drought stress does not have a significant correlation with the stomatal opening degree, stomatal length and width of GWu-2 (p<0.05).

The stomatal characteristics of GWu-2 under different water gradients (magnification of 500× and 2000×) are shown in Fig 3. The stomatal change trend under different water gradients is shown in Fig 4.

#### Correlation analysis between stomatal characteristic values of *Camellia oleifera* leaves and soil water content

Table 4 illustrates that soil water content was significantly positively correlated with stomatal opening degree of GWu-2 and negatively correlated with stomatal width. The correlation coefficient between soil water content and stomatal opening degree was the highest, at 0.93. The correlation coefficient between soil water content and stomata length was the lowest, at 0.06.

**Table 4:** Correlation analysis results of *Camellia oleifera* leaf stomatal characteristic indices and soil water content under different soil water gradients.

Index	Stomatal opening degree	Stomata length	Stomata width
Soil water	0.93*	0.06	–0.37

*Correlation is significant at the 0.05 level (2-tailed).

**Correlation is significant at the 0.01 level(2-tailed).

### Effects of drought stress on the leaf structure *of Camellia oleifera*

**Fig 5:**
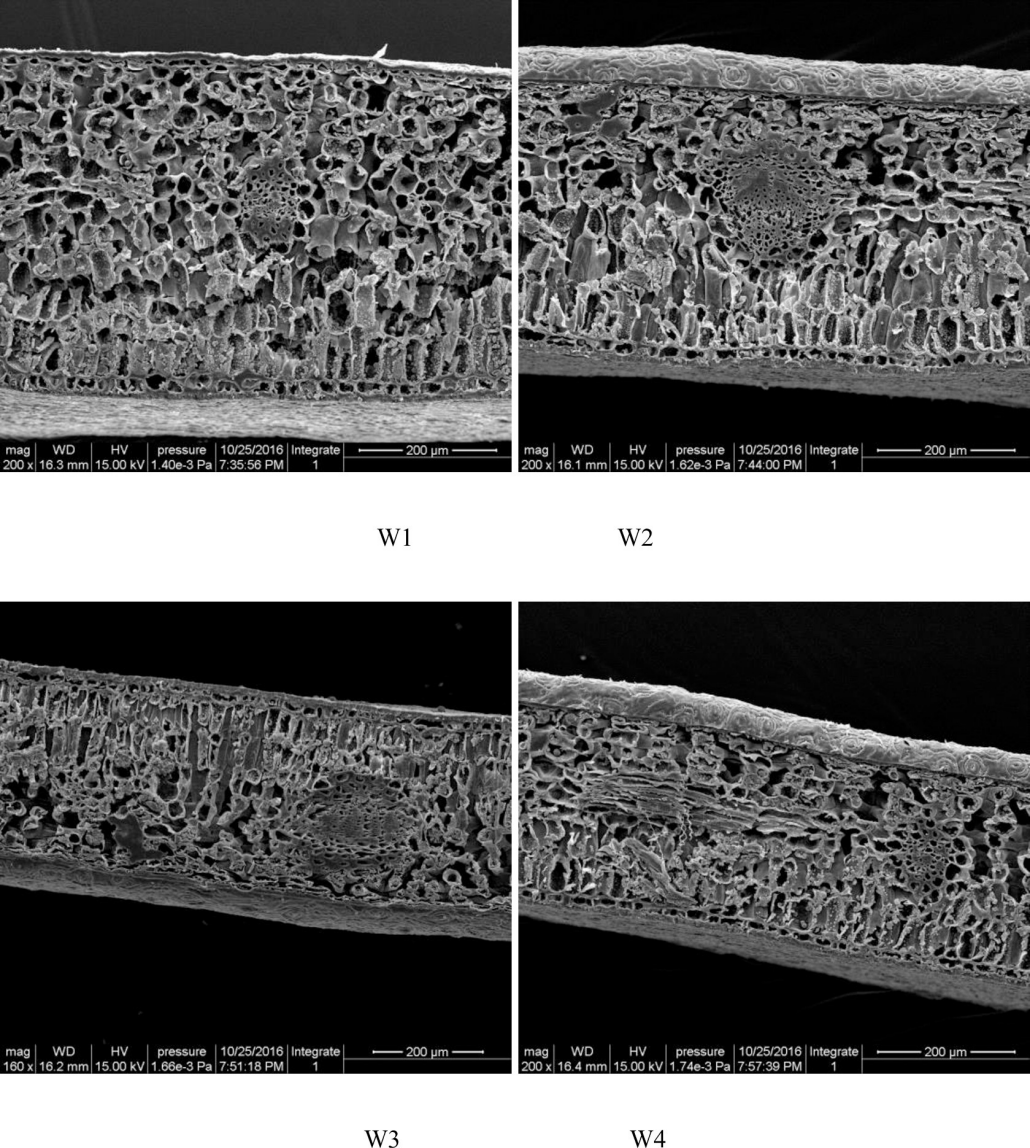
Electron micrographs of characteristics of *Camellia oleifera* leaves under different water gradients.

**Fig 6:**
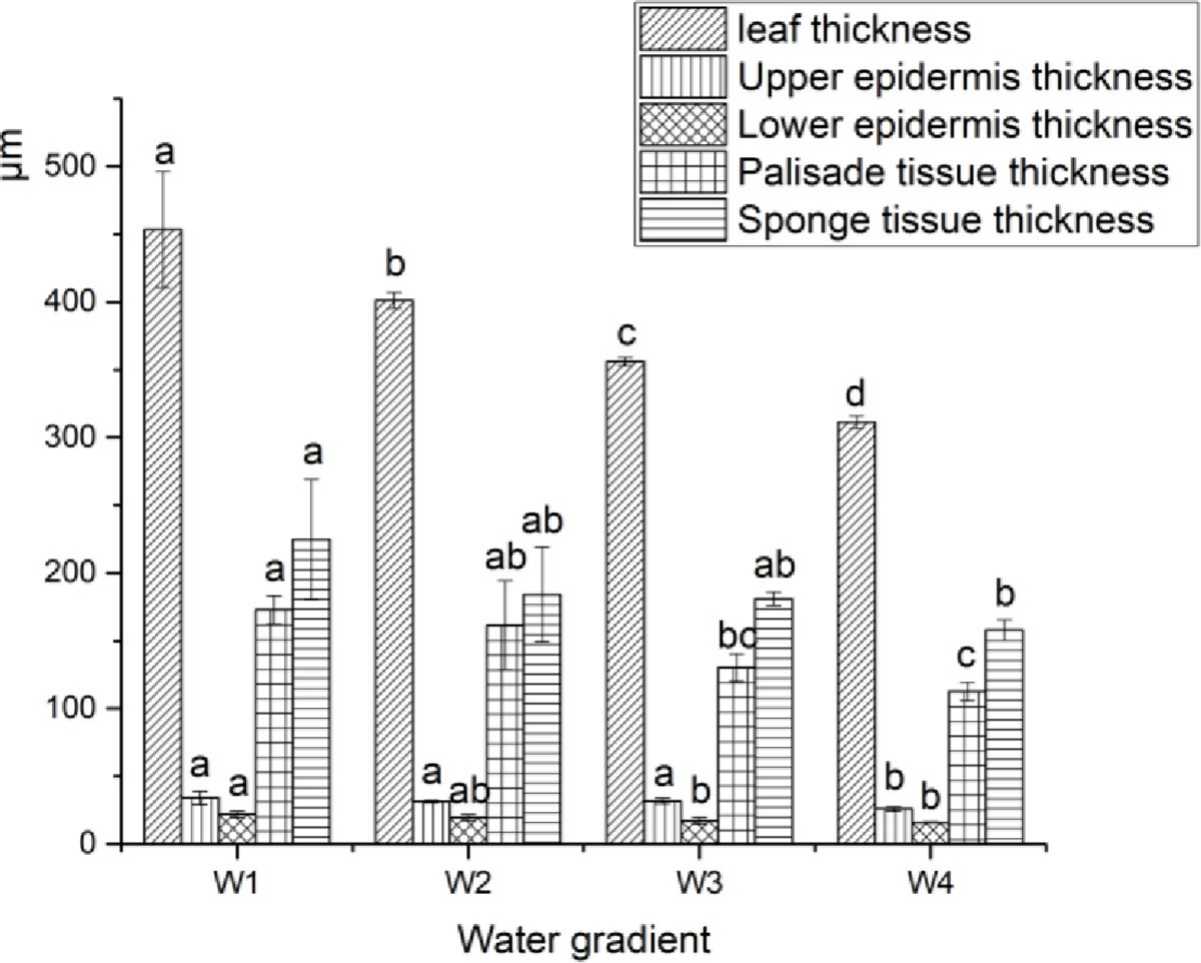
Thickness of leaf composition under different water gradients. W1: adequate water supply; W2: mild drought stress; W3: moderate drought stress; W4: severe drought stress. Each indicator value is an average value (Mean±SEs, n = 3).

Electron micrographs of the anatomical structure of GWu-2 under different water gradients (magnified 200× or 160×) are shown in Fig 5. The changes in leaf structure under different water gradients are shown in Fig 6.

Fig 5 indicates that drought stress had a certain effect on the arrangement and integrity of cells in *Camellia oleifera* leaves, and the leaves showed a tendency to decrease in thickness. Fig 6 illustrates that drought stress had significant effects on leaf, sponge tissue, palisade tissue, and lower and upper epidermal thickness of GWu-2 leaves (p<0.05), and with increased drought stress, these indicators showed decreasing trends and had the largest decreases under W4 treatment. Compared to W1 treatment, the leaf thicknesses of W2, W3, and W4 treatments were reduced by 11.5%, 21.5%, and 31.4%, respectively; the lower epidermis thicknesses were decreased by 11.4%, 21.6%, and 27.2%, respectively; the spongy tissue thicknesses were decreased by 6.6%, 24.5%, and 34.9%, respectively; and the palisade tissue thicknesses were decreased by 18.1%, 19.5%, and 29.8%, respectively.

#### Correlation analysis between *Camellia oleifera* leaf anatomical feature values and soil water content

Table 5 indicates that there were positive correlations between the anatomical characteristics of GWu-2 leaves and soil water content. Soil water content was significantly positively correlated with leaf, lower epidermis, and sponge tissue thickness. The correlation coefficient between soil water content and epidermis thickness was the largest, at 0.99. The correlation coefficient with upper epidermis thickness was the lowest, at 0.81.

**Table 5:** Correlation analysis between anatomical characteristics of *Camellia oleifera* leaves and soil water content under different water gradients.

Index	Leaf thickness	Upper epidermis thickness	Lower epidermis thickness	Palisade tissue thickness	Spongy tissue thickness	Leaf relative water
Soil water	0.97*	0.81	0.99*	0.92	0.97*	0.95

*Correlation is significant at the 0.05 level (2-tailed)

**Correlation is significant at the 0.01 level(2-tailed).

### Effects of drought stress on osmotic adjustment substances in *Camellia oleifera* leaves

**Fig 7:**
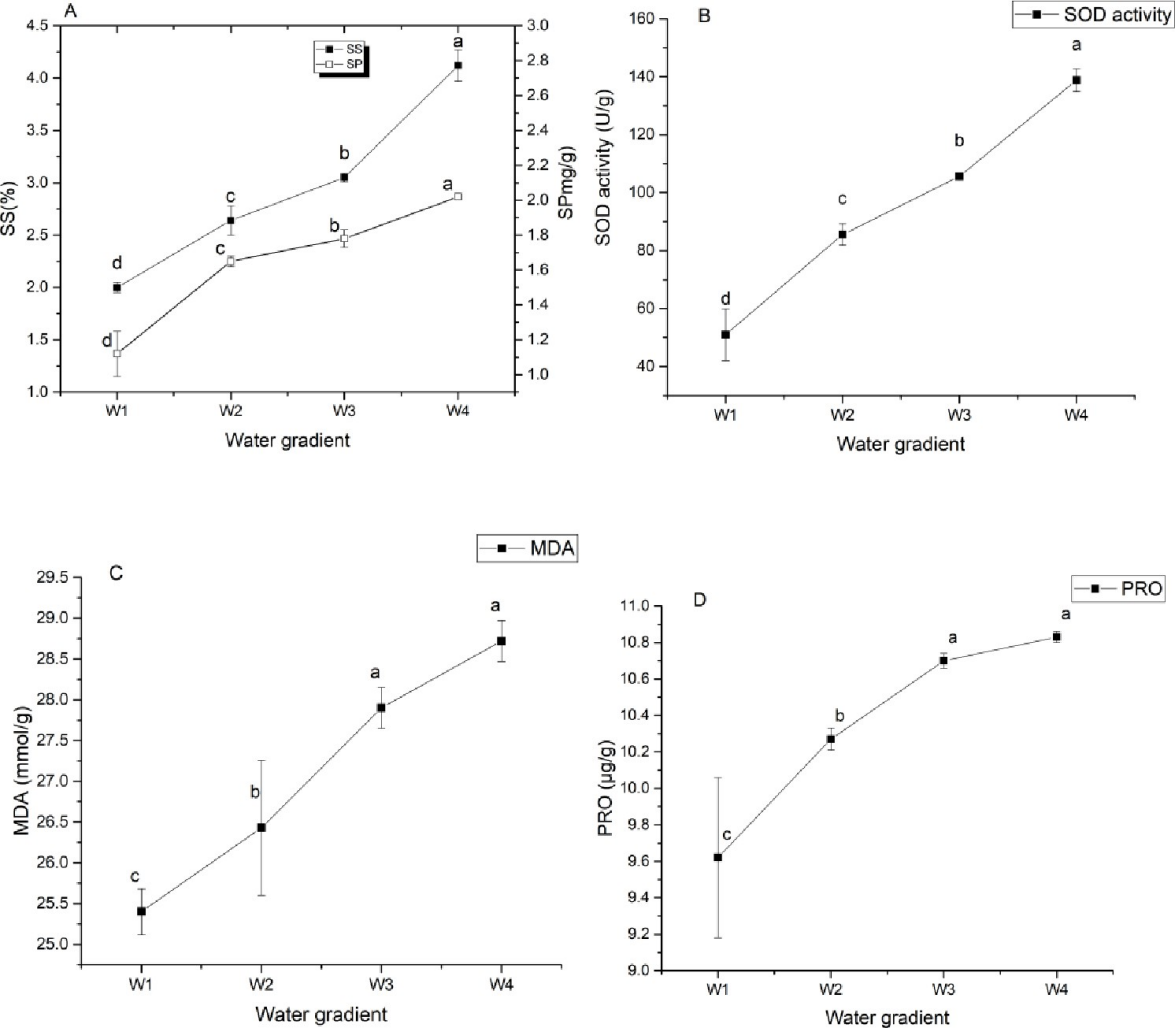
Osmotic adjustment substance content and superoxide dismutase (SOD) activity of *Camellia oleifera* leaves under different water gradients. W1: adequate water supply; W2: mild drought stress; W3: moderate drought stress; W4: severe drought stress; SS: soluble sugar; SP: soluble protein; MDA: malondialdehyde; PRO: proline; ABA: abscisic acid. Each indicator value is an average value (Mean±SEs, n = 3).

Fig 7 illustrates that the SS, SP, PRO, and MDA contents and SOD activity of leaves increased significantly with the increased drought stress. Among them, SS and SP contents and SOD activity were significantly increased under each water gradient (p<0.05), while under W4 treatment, there were no significant increases in PRO and MDA contents in GWu-2 leaves (compared with W3 treatment, p<0.05). With increased drought stress, compared with W1 treatment, the SS contents of W2, W3, and W4 treatments increased significantly, by 32.02%, 52.69%, and 106.05%, respectively; the SP contents increased by 47.45%, 59.20%, and 80.82%, respectively; and SOD activity increased by 67.86%, 107.14%, and 172.29%, respectively. The leaf PRO and MDA content changes increased to the maximum under W4 treatment, by 12.55% and 13.09%, respectively.

Table 6 indicates that there were high positive correlations between SS, SP, SOD, MDA, and PRO (greater than or equal to 0.9). The relative water content of the leaves was positively correlated with soil water content and negatively correlated with other indicators. The correlation between relative water content of the soil and other indicators was the same as the relative water content of the leaves.

**Table 6:** Correlation analysis results of osmotic adjustment substance in *Camellia oleifera* leaves under different water gradients.

Index	Soil water content	SS	SP	SOD	MDA	Relative water of leaves	PRO
Soil water content	1.00						
SS	–0.91	1.00					
SP	–0.99*	0.94	1.00				
SOD	–0.96*	0.99*	0.98*	1.00			
MDA	–0.96*	0.96*	0.95	0.98*	1.00		
Leaf relative water	0.95	–0.98*	–0.95	–0.99*	–0.99**	1.00	
PRO	–0.99**	0.90	0.98*	0.96*	0.97*	–0.95*	1.00

SS = soluble sugar; SP = soluble protein; SOD = superoxide dismutase; MDA = malondialdehyde; PRO = proline.

*Correlation is significant at the 0.05 level (2-tailed).

**Correlation is significant at the 0.01 level(2-tailed).

### Effects of drought stress on endogenous hormones in *Camellia oleifera* leaves

**Fig 8:**
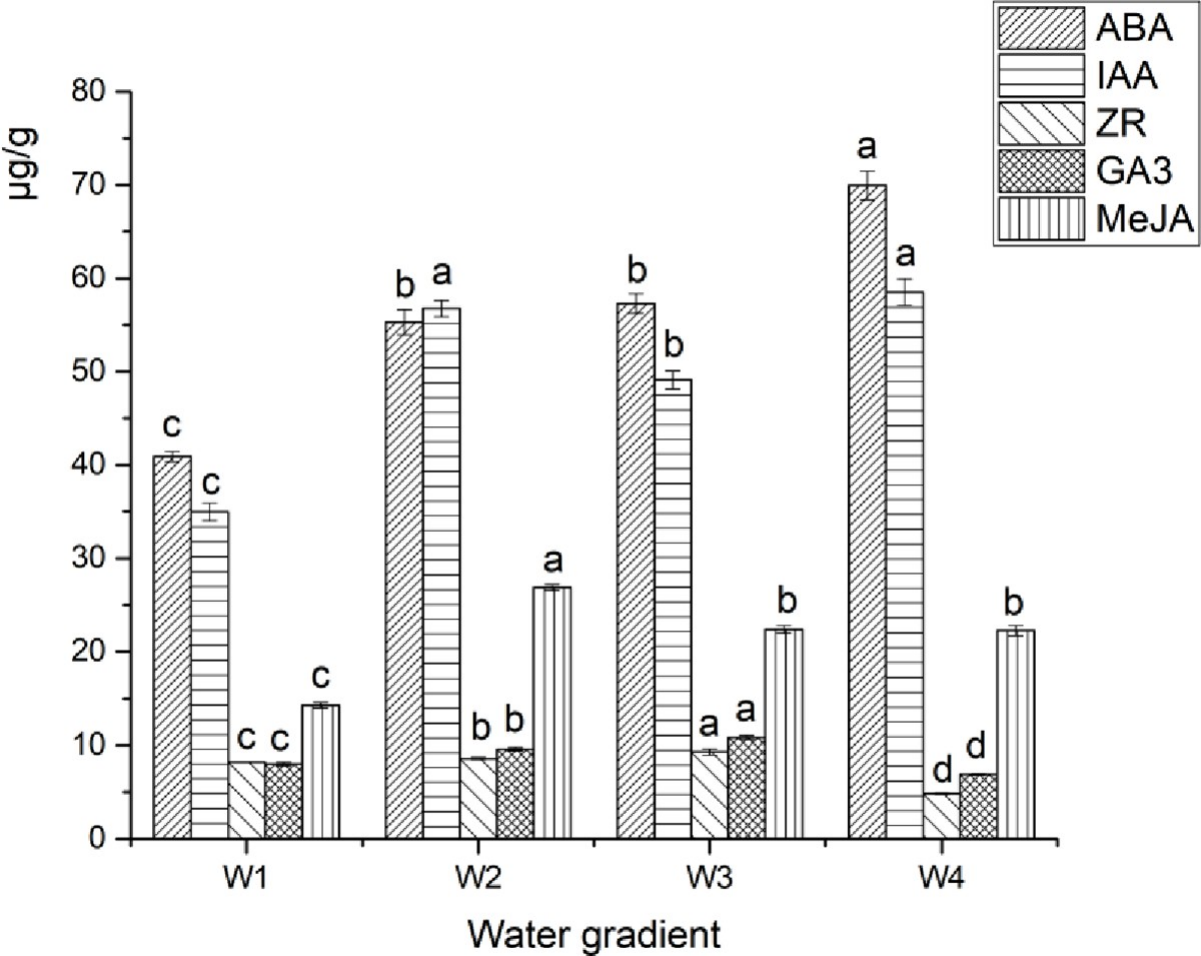
Changes in endogenous hormone content in *Camellia oleifera* leaves under different water gradients. Each indicator value is an average value (Mean±SEs, n = 3). ABA = abscisic acid; IAA = indole acetic acid; MeJA = methyl jasmonate; ZR = zeatin nucleoside; GA_3 =_ gibberellin.

Fig 8 illustrates that the ABA, IAA, ZR, GA3, and MeJA contents in GWu-2 leaves were significantly affected by drought stress. With increased drought stress, the ABA and IAA contents in GWu-2 leaves increased significantly (p<0.05). Compared with W1 treatment, under W2, W3, and W4 treatments, ABA contents increased by 35.21%, 40.06%, and 71.04%, respectively, and IAA contents increased by 62.35%, 40.45%, and 67.34%, respectively. GA3 content under W3 treatment reached the largest value (compared with W1 treatment), increasing by 35.27%. MeJA content under W2 treatment was the largest with the change of W1 treatment, increasing by 87.88%. However, ZR and GA3 contents significantly decreased under W3 treatments (compare to W4 treatment, p<0.05).

Table 7 indicates that SOD activity, SS content, and SP content were significantly positively correlated with ABA content, while relative water content, dry matter accumulation, SS content, SP content, and SOD activity of leaves were significantly negatively correlated with ABA content. Likewise, leaf relative water content and dry matter accumulation value were significantly positively correlated with ZR/GA3, while root-crown ratio, SS, SP and SOD activity were significantly negatively correlated with ZR/GA3.

**Table 7:** Correlation analysis results between hormone in *Camellia oleifera* leaves and growth index.

Index	ABA	IAA	MeJA	ZR	GA3	ZR/IAA	ZR/GA3
Leaf relative water	–0.95*	–0.72	–0.44	0.62	0.16	0.82	0.96*
Root-crown ratio	0.92	0.68	0.29	–0.86	–0.50	-0.89	-0.96*
Dry matter accumulation value	–0.95*	–0.73	–0.36	0.83	0.44	0.91	0.98*
Plant height growth value	–0.93	–0.89	–0.77	0.35	–0.16	0.80	0.90
Ground diameter growth value	–0.82	–0.47	–0.14	0.64	0.25	0.65	0.87
Number of dead leaves	0.82	0.55	0.12	–0.95	–0.68	-0.84	-0.88
Stomatal opening degree	–0.92	–0.68	–0.43	0.53	0.06	0.75	0.94
SS	0.97*	0.77	0.44	–0.75	–0.33	-0.91	-0.99**
SP	0.98*	0.90	0.70	–0.50	–0.01	-0.88	-0.96*
SOD	0.98*	0.81	0.54	–0.64	–0.18	-0.89	-0.99**
PRO	0.94	0.81	0.65	–0.39	0.12	-0.77	-0.92

ABA = abscisic acid; IAA = indole acetic acid; MeJA = methyl jasmonate; ZR = zeatin nucleoside; GA3 = gibberellin.

*Correlation is significant at the 0.05 level (2-tailed).

**Correlation is significant at the 0.01 level (2-tailed).

## Discussion

### Effect of drought stress on the growth of *Camellia oleifera*

Plant roots play an important role in response to drought stress. Under drought stress conditions, plant roots can quickly adjust to cope with water shortage [25]. In this experiment, as the degree of drought increased, especially under W4 treatment, the dry matter accumulation and total root length of GWu-2 were significantly decreased (Fig 1A and 1C), indicating that under drought stress, the growth of both above-ground and underground parts of GWu-2 was affected. However, the number of lateral roots and the root-crown and root-plant ratios of GWu-2 did not decrease significantly (Fig 1B and 1C), indicating that under drought stress, although the growth of GWu-2 was restrained, compared with the aerial parts, *Camellia oleifera* gives priority to the normal growth of the root system to maintain the contact area with soil and to obtain the necessary water [12], which is consistent with other studies [26]. Correlation analysis (Table 3) showed that the number of lateral roots was negatively correlated with soil water content, and the correlation coefficient reached –0.91, further confirming this conclusion.

The external morphological characteristics of the leaves can directly reflect the drought stress response of the plant, and the external morphological characteristics of leaves are significantly related to the degree of water deficit [11]. In the present study, the increased degree of drought had a significant influence on the normal physiology and growth of leaves, and the leaf thickness and relative water content of GWu-2 were significantly decreased (Figs 2B and 6). Moreover, under W4 treatment, the number of dead leaves significantly increased (Fig 2A), and the damage to the GWu-2 leaf structure became more serious as the degree of drought increased (Fig 5). These responses to drought stress indicate that the growth of GWu-2 leaves was greatly affected by drought stress. In addition, ABA content will cause leaf detachment and accelerate senescence [27]; thus, the increasing of ABA content (Fig 8) may be one of the reasons for the increased dead leaves of GWU-2 under W4 treatment.

### Effect of drought stress on *Camellia oleifera* stomata

As important organs for gas exchange in plants, the stomata are mainly distributed on the lower epidermis of leaves, which is the main channel for water vapor loss and inflow and outflow of CO_2_, and stomata may close due to the effects of drought [28]. As Fig 3 shows, with increased drought stress, the stomatal opening degree decreased; under W4 treatment, the stomata of GWu-2 were damaged and subsided, and the epidermal cells became dried, indicating that drought has a adverse effect on the stomata of GWu-2. However, drought stress did not have a significant influence on the stomatal length and width of GWu-2 (Fig 4, p<0.05), indicating that GWu-2 may reduce water transpiration by adjusting the stomatal opening degree under drought stress [15]. Correlation analysis (Table 4) showed that the correlation coefficient between soil water content and stomatal opening degree was significantly positive, which further confirmed this conclusion.

### Effect of drought stress on *Camellia oleifera* leaf osmotic adjustment substances, SOD and MDA

SS, PRO and SP are important osmotic adjustment substances in plants. Under drought stress, the contents of these osmotic adjustment substances in plants usually increase, thus maintaining the osmotic pressure and normal metabolism [29]. In this experiment, with increased drought stress, the SS, PRO and SP contents of GWU-2 leaves increased significantly (Fig 7A and 7D), consistent with the reports about rice [30] and winter wheat [31]. The changes in SS, PRO, and SP content in GWu-2 leaves verified that regulation of the content of osmotic substances is one of the physiological mechanisms of *Camellia oleifera* adaptation to drought stress. However, the responses of different kinds of osmotic adjustment substances to drought stress may be different. Some scholars have pointed out that fructose and sucrose are more effective in drought resistance than PRO [32]. In this study, the contents of SS and SP under W4 drought stress were significantly increased relative to W3, while PRO content was not increased, indicating that SS, PRO, and SP may have different contributions to the drought resistance of *Camellia oleifera*.

SOD is a plant antioxidant enzyme [33], and MDA is one of the most important products of membrane lipid peroxidation. The changes of their content under drought stress can reflect the level of peroxidation of lipid membranes and the degree of damage to cell membranes as well as the drought resistance of plants. In this experiment, with increased drought stress, the SOD activity and MDA content significantly increased (Fig 7B and 7C), indicating that GWu-2 resists drought stress by increasing leaf MDA content and SOD activity, consistent with other research findings [34,35]. The negative correlations between soil water content and SOD activity and MDA content of GWu-2 further confirmed this conclusion (Table 6).

Table 6 indicates that SOD activity and the SS, SP, PRO, and MDA contents of the leaves were positively correlated and were significantly negatively correlated with the relative water content of the leaves, indicating that the osmotic adjustment and antioxidant effects of GWu-2 in response to drought stress were synergistic, consistent with previous studies [36].

### Effect of drought stress on *Camellia oleifera* leaf endogenous hormones

Changes in the contents of various endogenous hormones in plants have an important impact on plant growth [37, 38]. Among them, ABA and MeJA are hormones that are sensitive to stress in plants. Their effective accumulation in drought stress environments plays an important role in regulating plant growth and stomatal opening or closing and enhancing drought tolerance [39–41]. In this experiment, leaf ABA, IAA and MeJA contents were significantly increased with the increase of drought stress and were negatively correlated with the stomatal opening degree, on the contrary, ZR/IAA and ZR/GA3 were positively correlated with the stomatal opening degree (Fig 8 and Table 7), indicating that *Camellia oleifera* by controlling ABA, IAA, and MeJA content as well as the ratio of hormones to control stomatal opening, consistent with previous studies [42,43,44].

IAA, ZR, and GA3 are important hormones for plant growth [45]. Additionally, changes in the contents of endogenous hormones in plants have important effects on plant growth and are affected by the interactions of multiple hormones [46]. In this study, we observed that the IAA, ZR, and GA3 contents in GWu-2 leaves increased significantly with increased drought stress, consistent with the reports by Ding and Chen [47,48]. However, compared with W3 treatment, ZR and GA3 contents decreased under W4 treatment, indicating that different hormones respond to drought differently and may play a different role in regulating growth and drought resistance [49]. Although there were no significant correlations between physiological indicators and IAA, ZR and GA3 contents, significant correlations between ABA, ZR/GA3 ratio and physiological indicators were observed (Table 7), indicating that the drought resistance of *Camellia oleifera* may be regulated by hormone secretion and the proportions of different hormones [48].

## Conclusions

In this study, *Camellia oleifera* GWu-2 was used as the research subject, and a total of four soil water gradients were set up, including one normal water supply group and three levels of water shortage control groups. The growth characteristics, permeating substance content in leaf, leaf enzyme activity, and leaf hormone contents under different water gradients were observed. The observation results were analyzed, and the response mechanism of *Camellia oleifera* GWu-2 to drought stress was discussed. The main results are as follows:

We found that, with increased drought stress, the normal growth of GWu-2 was affected, the dry matter accumulation of GWu-2 was significantly decreased and was positively correlated with soil water content, and the root growth was not significantly affected. Likewise, the leaf thickness and relative water content of GWu-2 leaves decreased, and the number of dead leaves increased significantly under severe drought stress. The stomata were also affected by drought, as the stomatal opening degree decreased as the degree of drought increased, and as the stomata were damaged and subsided, and the epidermal cells became dried under severe stress.

SOD activity and SS, SP, PRO, and MDA contents in GWu-2 leaves increased significantly and were negatively correlated with soil water content. ABA, MeJA, GA3, ZR and IAA contents in GWu-2 leaves increased under drought stress, but different hormones respond differently to drought stress and play different roles in the growth regulation and drought resistance of GWu-2. We concluded that the drought-resistance mechanism of GWu-2 was controlled by maintaining root growth to obtain the necessary water, increasing the content of osmotic substances of leaves to maintain water holding capacity, reducing the transpiration of water by closing the stomata and reducing the damage caused by drought by increasing the activity of SOD. These changes are largely controlled by hormone secretion.

## Supporting information

S1 Data (RAR)
